# CD98 Increases Renal Epithelial Cell Proliferation by Activating MAPKs

**DOI:** 10.1371/journal.pone.0040026

**Published:** 2012-06-29

**Authors:** Nada Bulus, Chloe Feral, Ambra Pozzi, Roy Zent

**Affiliations:** 1 Department of Medicine (Division of Nephrology), Vanderbilt University Medical Center, Tennessee, United States of America; 2 Department of Cancer Biology, Vanderbilt University Medical Center, Tennessee, United States of America; 3 Department of Cell and Developmental Biology, Vanderbilt University Medical Center, Tennessee, United States of America; 4 Department of Medicine, Veterans Affairs Hospital, Nashville, Tennessee, United States of America; 5 Institute for Research on Cancer and Aging, University of Nice Sophia-Antipolis, Centre Antoine Lacassagne, Nice, France; Emory University, United States of America

## Abstract

CD98 heavy chain (CD98hc) is a multifunctional transmembrane spanning scaffolding protein whose extracellular domain binds with light chain amino acid transporters (Lats) to form the heterodimeric amino acid transporters (HATs). It also interacts with β1 and β3 integrins by its transmembrane and cytoplasmic domains. This interaction is proposed to be the mechanism whereby CD98 mediates cell survival and growth via currently undefined signaling pathways. In this study, we determined whether the critical function of CD98-dependent amino acid transport also plays a role in cell proliferation and defined the signaling pathways that mediate CD98-dependent proliferation of murine renal inner medullary collecting duct (IMCD) cells. We demonstrate that downregulating CD98hc expression resulted in IMCD cell death. Utilizing overexpression studies of CD98hc mutants that either lacked a cytoplasmic tail or were unable to bind to Lats we showed that CD98 increases serum-dependent cell proliferation by a mechanism that requires the CD98hc cytoplasmic tail. We further demonstrated that CD98-dependent amino acid transport increased renal tubular epithelial cell proliferation by a mechanism that does not require the CD98hc cytoplasmic tail. Both these mechanisms of increased renal tubular epithelial cell proliferation are mediated by Erk and p38 MAPK signaling. Although increased amino transport markedly activated mTor signaling, this pathway did not alter cell proliferation. Thus, these studies demonstrate that in IMCD cells, the cytoplasmic and extracellular domains of CD98hc regulate cell proliferation by distinct mechanisms that are mediated by common MAPK signaling pathways.

## Introduction

The heterodimeric amino acid transporters consist of a type II transmembrane protein heavy chain and a light chain linked by an extracellular disulfide bridge [1], [2]. The heavy chain subunits rBAT and CD98hc (also called 4F2hc) heterodimerize with a number of light chain amino acid transporters [3], [4]. The principal function of the heavy chain is to localize the heterodimer to either the apical or basolateral aspect of the cell [1], [2]. CD98 is expressed in all cell types with the exception of platelets and its highest levels of expression are in the tubules of the kidney and the gastrointestinal tract [1], [2], [5], where it plays a critical role in the vectorial transport of amino acids across a polarized epithelium. CD98 is required for normal development in mammals and deletion of CD98hc results in early embryonic lethality in mice [6].

CD98hc heterodimerizes with one of the light chains Lat-1, Lat2, y^+^Lat-1, y^+^Lat2 and xCT [7]–[16] and is required for the surface expression of the heterodimers. These transporters mediate Na^+^-independent transport of large neutral amino acids (e.g. leucine) and/or Na^+^-dependent co-transport of positively charged amino acids (e.g. arginine) and neutral amino acids [1], [2]. In addition to the light chain, CD98hc associates with β integrins [17]–[26]. This association is important for altering integrin affinity and integrin dependent signaling resulting in alterations in cell differentiation, proliferation, aggregation, adhesion, migration and malignant transformation [17]–[21], [23]–[26]. The physiological relevance of CD98 function was investigated in mice by either deleting or overexpressing CD98hc [23], [24], [27]–[30]. Constitutive deletion of CD98hc resulted in early embryonic lethality [6]. Specifically deleting CD98hc from lymphocytes [27], [28] and vascular smooth muscle [29] decreased proliferation of both cell types and altered adaptive humoral immunity or resulted in abnormal vessel repair in the organ specific null mice. CD98hc overexpression in the gastrointestinal epithelium induced tumorigenesis by causing barrier dysfunction and stimulating cell proliferation, whereas CD98hc deletion resulted in an attenuated inflammatory response as well as resistance to DSS-induced colitis and colitis-associated tumorigenesis [30]. Together these in vivo data emphasize the important role CD98 plays in regulating cell proliferation in multiple cell types.

The mechanism whereby CD98 modulates cell proliferation is not fully understood. Increased amino acid transport increases cell proliferation [31]–[33], however mechanisms other than this also probably play a role in CD98-dependent proliferation as mutants of CD98hc unable to associate with amino acid transporters can induce malignant transformation of NIH3T3 cells [34]. In addition, CD98/β1 integrin interactions which have no effect on amino acid transport have been shown to be important in modulating CD98-dependent transformation [18], survival [23], [27], [35], proliferation [23], [27], [29], [30], [35], adhesion [18], [26], migration [18], [26] and tubule formation [26]. PI3-kinase [18], [26], Rho A [23] and focal adhesion kinase [18], [26] signaling have been implicated in mediating CD98/β1 integrin-dependent cell spreading, migration, transformation and survival. The relative roles of CD98-dependent amino acid transport and other as yet undefined signaling pathways on cell proliferation are currently unclear.

Based on the *in vivo* and in *in vitro* data showing a requirement for CD98 for cell survival and proliferation, we investigated the mechanisms whereby CD98 regulates these cellular processes in polarized renal epithelial cells. We demonstrate that CD98hc expression is required for these cells to survive, as deleting or downregulating CD98hc expression results in cell death. Utilizing overexpression studies of mutants of CD98hc and Lat-1 we show that CD98 increases cell proliferation by two distinct mechanisms that signal through MAPKs. One requires the CD98hc cytoplasmic tail and is independent of changes in amino acid transport. The second is due to increased CD98-dependent amino acid transport and does not require the CD98hc cytoplasmic tail. Thus CD98 modulates cell proliferation by two distinct mechanisms, namely the induction of signaling via its cytoplasmic tail and its ability to increase amino acid transport into cells.

## Results

### CD98 is Required for Inner Medullary Collecting Duct (IMCD) Cell Survival

**Figure 1:**
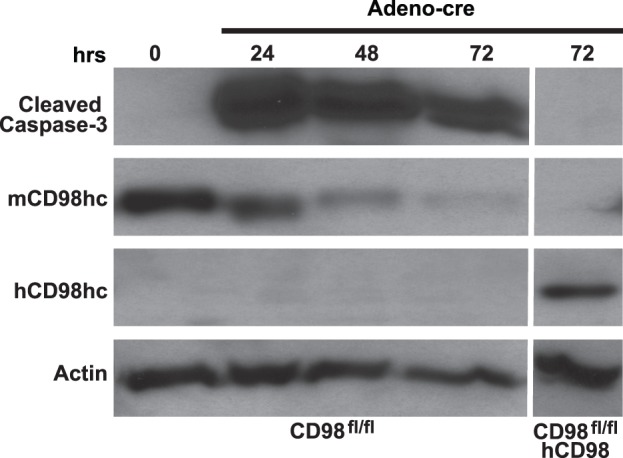
CD98 is required for IMCD cell survival. CD98^fl/fl^ IMCD cells or CD98^fl/fl^ IMCD cells reconstituted with human CD98 (CD98^fl/fl^hCD98) were infected with adeno-cre virus after which they were harvested at different time points and immunoblotted for cleaved caspase-3, mouse CD98hc, human CD98hc or actin.

Consistent with previous reports demonstrating that CD98 expression increases cell proliferation [23], [27], [29], [30], [35], we observed that IMCD cells (which are derived from the kidney collecting ducts) overexpressing CD98 proliferated more rapidly than wild type IMCD cells under normal culture conditions. To define the molecular mechanisms of this increased cell proliferation, we initially attempted to downregulate CD98hc in IMCD cells using 3 different hairpin siRNAs expressed by lentiviruses. Although we successfully downregulated CD98hc expression using all 3 lentiviruses, the cells died within a week (data not shown). We next attempted to generate IMCD cells from CD98hc floxed mice and delete the gene *in vitro* using cre recombinant adenovirus. While we were able to obtain CD98hc^fl/fl^ cells, we were unable to obtain CD98hc-null cells, as they also died within a week (data not shown). To demonstrate the requirement for CD98hc for IMCD cell survival, we transfected CD98hc^fl/fl^ cells with a human CD98hc cDNA and then deleted the floxed gene in vitro with adeno cre. In figure 1, we show that CD98^fl/fl^ cells treated with cre show increased caspase-3 activation and this correlates with the degree of downregulated mouse CD98hc protein. In contrast, we did not detect any caspase-3 activation in CD98^fl/fl^ cells that expressed human CD98hc following cre treatment. All the cre- infected CD98hc^fl/fl^ IMCD cells died eventually, while the reconstituted cells survived and continued proliferating. Thus, deleting CD98hc results in significant IMCD cell apoptosis suggesting these cells require CD98hc for survival.

### The Cytoplasmic Tail of CD98 is Required to Induce Serum Dependent Cell Proliferation

**Figure 2:**
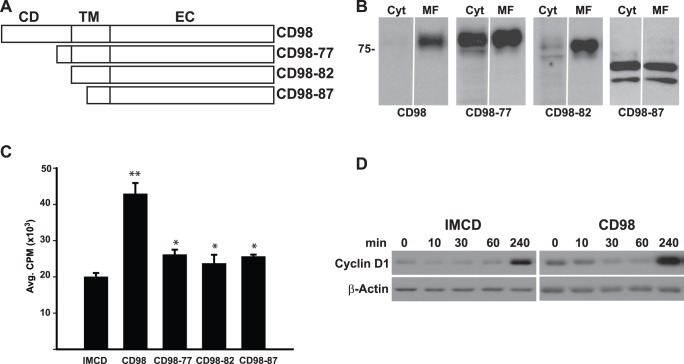
The cytoplasmic tail of CD98 is required for serum-dependent cell proliferation. (A) Schematic representation of the CD98 constructs used for CD cell transfection. CD, cytoplasmic domain; TM, transmembrane domain; EC, extracellular domain. (B) Full-length CD98hc and its truncation mutants are expressed in membrane fractions of IMCD cells. IMCD cells expressing CD98hc or the different truncation mutants were fractionated into cytoplasmic (Cyt) Triton X-100–soluble and – insoluble membrane fractions (MF). These fractions were subjected to SDS-PAGE and immunoblotted with an antibody directed against human CD98hc. (C) CD98hc induces cell proliferation in serum. IMCD were plated onto 96-well plates, serum deprived for 24 hours then treated for 16 hours with ^3^H-Thymidine in media containing 1% serum. ^3^H-Thymidine incorporation was determined as described in Experimental Procedures. Values are the mean ± s.e.m. of a representative experiment performed in eight replicates. (*) indicates statistically significant differences (p<0.05) between CD98 and deletion mutants. (**) denotes a statistically significant difference between CD98 and IMCD. (D) Time course of Cyclin D1 expression. Sub-confluent cells were serum deprived for 24 hours and then treated with complete medium. Lysates were isolated at the time points indicated and analyzed by SDS-PAGE.

Due to our failure to establish CD98hc-null cell populations we utilized previously generated IMCD cells that overexpress full length (CD98) and mutants of human CD98hc [26] to define how CD98 regulates cell proliferation. The mutants (Figure 2A) either lacked the first 77 amino acids of the cytoplasmic tail (CD98-77), the full putative cytoplasmic tail (CD98-82) or the cytoplasmic tail as well as the N-terminal five amino acids (WALLL) of the transmembrane domain (CD98-87), which we have shown to be critical for CD98 to interact with the β1 integrin and regulate integrin dependent cell adhesion, migration and tubulogenesis [26]. Equal expression of these mutants in the IMCD cells was verified by flow cytometry with an antibody directed against the extracellular domain of human CD98hc, which demonstrates that the CD98hc is expressed on the cell surface [26]. We further showed that all of these mutants are expressed in membranes by performing western blot analysis with an antibody directed against human CD98hc on cytosolic and membrane fractions derived from the IMCD cells (Figure 2B).

We defined the role of CD98 on IMCD cell proliferation in cells grown in complete medium containing low levels (1%) of serum (Figure 2C). CD98 cells showed a 2 fold increase in cell proliferation relative to IMCD cells and consistent with this result, there was increased cyclin D1 expression in CD98 cells 4 hours after the addition of complete medium (Figure 2D). By contrast, there was only approximately a 35% increase in proliferation in the CD98-77, CD98-82 or CD98-87 deletion mutants (Figure 2C). These results suggest that increased CD98hc expression enhances cell proliferation in complete medium by a mechanism that requires the 77 amino acid cytoplasmic tail and another that does not.

### CD98 Regulates Serum-dependent Cell Proliferation by Increasing Erk and p38 MAPK Signaling

**Figure 3:**
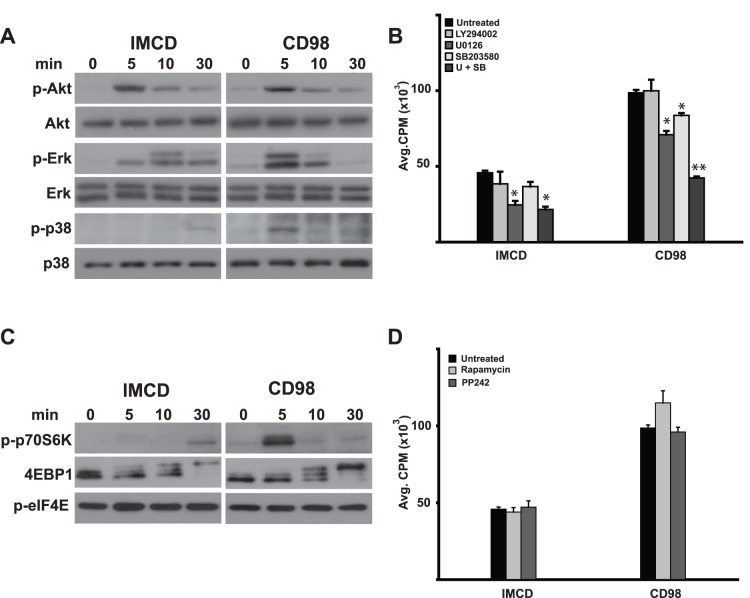
CD98 regulates serum dependent cell proliferation by increasing Erk and p38 MAPK signaling. (A, C) CD98hc expression increases MAPK, Akt and mTor signaling in response to serum. Subconfluent cells were serum deprived for 24 hours then treated with complete medium. Lysates were prepared at the time points indicated and analyzed by western blot with the antibodies indicated. (B, D) MAPK regulates CD98hc-dependent IMCD cell proliferation. Cells seeded onto 96-well plates were serum deprived for 24 hours, pretreated with inhibitors prior to addition of ^3^H-Thymidine in complete medium as described in Experimental Procedures. U+SB denotes concomitant treatment with the MEK inhibitor U0126 (U) and the p38 MAPK inhibitor SB203580 (SB). (*) indicates statistically significant differences (p<0.05) between treated and untreated cells. (**) denotes statistically significant differences between U+SB versus either inhibitor alone.

Complete medium, which includes fetal calf serum, increases cell proliferation by altering many different signaling pathways. Because the data presented above suggests that the CD98hc cytoplasmic tail regulates cell proliferation and CD98hc has been demonstrated to activate specific integrin-dependent signaling pathways [17]–[19], we initially defined whether there were differences in serum-dependent (complete medium with 10% serum) activation of PI3K, Erk and p38 MAPK signaling between IMCD cells and CD98 cells. In contrast to the changes seen in integrin-dependent signaling where the PI3K pathway was activated [18], [26], there was no change in p-Akt signaling between the wild type and CD98 cells. There was however earlier and more pronounced Erk and p38 MAPK activation in the CD98 cells (Figure 3A). To define whether this increased signaling altered cell proliferation, thymidine incorporation assays were performed in low serum medium (1%) in the presence and absence of the PI3K inhibitor LY294002, the MEK inhibitor U0126 and the P38 MAPK inhibitor SB203580 (Figure 3B). IMCD cell proliferation was decreased by 40% with the MEK inhibitor; however CD98 cell proliferation was significantly decreased by both the MEK (40%) and p38 MAPK (25%) either added alone or in combination.

Because overexpression of CD98 increases amino acid transport and amino acids are well known to stimulate the mTor pathway, we defined the role of this pathway in modulating proliferation of CD98 cells. We assessed whether the mTor pathway was activated by defining the phosphorylation state of p70S6K and 4EBP1, both of which are downstream of mTorc1. Adding complete medium with 10% serum stimulated phosphorylation of p70S6K only in CD98 cells and, although the phosphorylation pattern of 4EBP1 was similar in both cell lines, there was a more robust signal in CD98 cells 30 minutes after stimulation (Figure 3C). Despite increased mTOR downstream signaling in CD98 cells, addition of the mTor inhibitors Rapamycin or PP242 did not alter cell proliferation (figure 3D). These results suggest that proliferation of IMCD cells, which is increased in cells overexpressing CD98, is dependent on Erk and p38 MAPK but not mTor or PI3K activation.

### IMCD Cell Proliferation is Increased in Response to CD98-dependent Enhanced Amino Acid Transport

**Figure 4:**
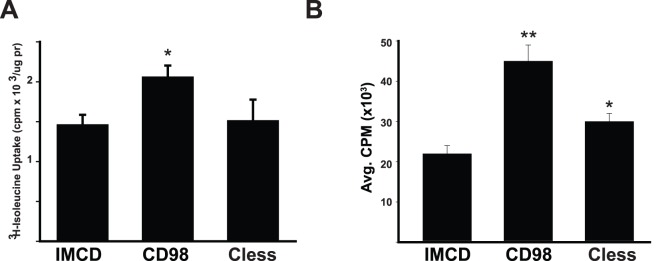
IMCD cell proliferation increases in response to enhanced amino acid transport. (A) CD98 expression increases isoleucine transport. ^3^H-Isoleucine uptake was performed as described in Experimental Procedures. Cless cells are IMCD transfected with a CD98 mutant unable to interact with Lats. (*) denotes statistically significant differences (p<0.05) between IMCD and CD98 cells. (B) Transfected IMCD cells were plated onto 96-well plates, serum deprived for 24 hours then treated for 16 hours with ^3^H-Thymidine in media containing 1% serum. ^3^H-Thymidine incorporation was determined as described in Experimental Procedures. Values are the mean ± s.e.m. of a representative experiment performed in eight replicates. (*) indicates statistically significant differences (p<0.05) between IMCD and Cless cells. (**) denotes statistically significant differences between CD98 and Cless cells.

CD98hc interacts with the Lat transporters via its extracellular domain and increases CD98-dependent amino acid transport. To define the role of amino acid transport by CD98 on cell proliferation we generated IMCD cells that overexpressed Cless, a previously defined mutant of CD98hc where the two cysteines in the extracellular domain are mutated making it unable to interact with the HAT light chains [36]. This population of cells expressed Cless at the same level as CD98 as verified by flow cytometry (data not shown). As expected, overexpressing CD98 alone caused a 40% increase in isoleucine transport relative to IMCD cells which was not seen in the Cless cells (Figure 4A). When proliferation in complete medium with 1% serum was determined in the Cless IMCD cells, there was only a 50% increase, while there was over a 2 fold increase in the CD98 cells compared to IMCD cells (Figure 4B). These data suggest that the extracellular domain of CD98 increased proliferation in the CD98 cells by inducing additional amino acid transport.

**Figure 5:**
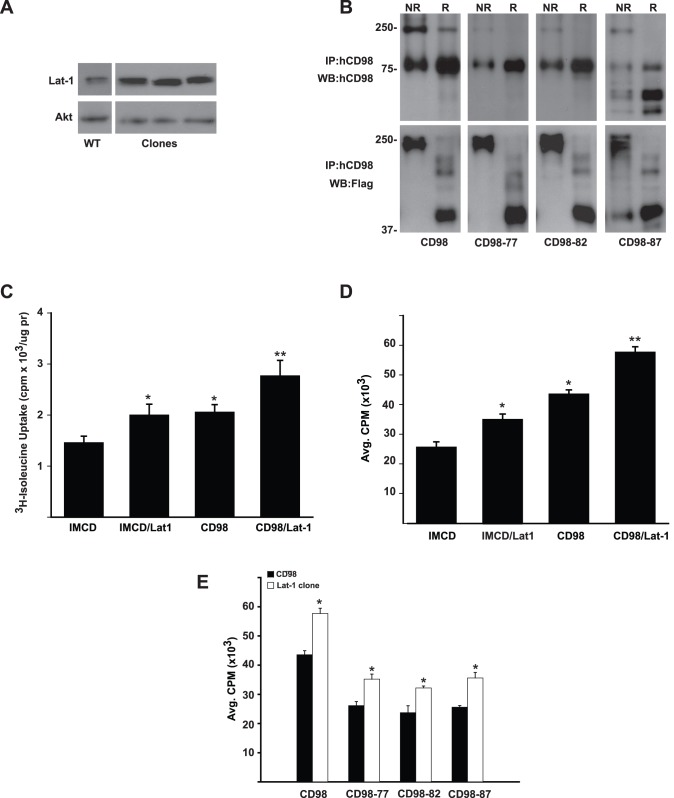
CD98hc and Lat-1 increase cell proliferation. (A, B) Overexpressed Lat-1 interacts with CD98hc and CD98hc deletion mutants. **(A)** Western blot analysis of clones of IMCD cells transfected with Lat-1. Total Akt was used as a loading control. (B) Cells overexpressing CD98 or CD98 deletion mutants as well as Flag-tagged human Lat-1 were lysed and immunoprecipitated with a human specific CD98 antibody. Non-reduced (NR) and reduced (R) immunoprecipitates were separated by SDS-PAGE and immunoblotted with either a human specific CD98 antibody (upper panel) or Flag antibody (lower panel). (C, D) Co-expression of CD98hc and Lat-1 increases amino acid transport and cell proliferation. (C) ^3^H-Isoleucine uptake was performed as described in Experimental Procedures. IMCD/Lat-1cells are IMCD cells transfected with Lat-1 only and CD98/Lat-1 cells were the Lat-1 cells that were further transfected with CD98 and selected by flow cytometry. (*) denotes statistically significant differences (p<0.05) compared to IMCD cells whereas (**) indicates significant differences compared to CD98 cells. (D) Transfected cells were plated onto 96-well plates, serum deprived for 24 hours then treated for 16 hours with ^3^H-Thymidine in media containing 1% serum. ^3^H-Thymidine incorporation was determined as described in Experimental Procedures. Values are the mean ± s.e.m. of a representative experiment performed in eight replicates. (*) indicates statistically significant differences (p<0.05) compared to IMCD cells. (**) denotes a statistically significant difference between CD98 and CD98/Lat-1. (E) Cells expressing CD98 deletion mutants and Lat-1 proliferated less than CD98/Lat-1 cells. IMCD cells overexpressing Lat-1 were transfected with either CD98 or CD98 deletion mutant constructs described in Fig.1. Thymidine incorporation was determined as described above. Values are the mean ± s.e.m. of a representative experiment performed in eight replicates. (*)indicates statistically significant differences (p<0.05) between double transfectants and cells transfected with CD98 or deletion mutants only.

To further explore the effects of amino acid transport of HATs on IMCD cell proliferation, we generated IMCD cells that overexpressed the amino acid transporter Lat-1 in conjunction with CD98hc. We utilized a Flag-tagged Lat-1 for these over-expression studies. Lat-1 is endogenously expressed in IMCD cells (Figure 5A) and we were able to generate multiple clones of IMCD cells (3 shown) which overexpressed Lat-1. We subsequently overexpressed CD98 or the truncation mutants of CD98-77, CD98-82 and CD98-87 into the Lat-1 overexpressing cells and sorted for equal expression by flow cytometry (data not shown). To verify that Lat-1 heterodimerized with CD98hc or the mutants, we immunopreciptated CD98hc from the different IMCD cell populations and immunoblotted the precipitates with antibodies directed against either CD98hc (Figure 5B, upper panel) or Flag (Figure 5B, lower panel). It is clear that Lat-1 was part of the HAT complex in non-reducing conditions and that it became disassociated from CD98hc under reducing conditions. Thus, the HAT complex was formed by CD98hc and all the mutants.

We initially examined the effect of overexpressing Lat-1 in the IMCD cells. Like CD98 cells, the Lat-1 expressing cells had a small but significant (40%) increase in isoleucine transport when compared to IMCD cells, while expressing both CD98 and Lat-1 resulted in an 2 fold increase in isoleucine transport (Figure 5C). Consistent with this increase in amino acid transport there was a 40% increase in proliferation of IMCD/Lat-1 cells relative to wildtype IMCD cells in low serum medium. Furthermore, cells overexpressing Lat-1 increased CD98 cell proliferation by an additional 38% such that these cells proliferated 2.4 fold more than IMCD cells (Figure 5D). Similar to CD98 cells, expressing the Lat-1 transporter increased cell proliferation of cells of CD98-77, CD98-82 and CD98-87 cells by 40% in low serum medium (Figure 5E). These studies further confirm that CD98-dependent amino acid transport increased proliferation by a mechanism that does not require the CD98hc cytoplasmic tail.

### Increased Transport of Amino Acids by CD98 Induces Activation of Erk and p38 MAPK Pathways which Enhance Cell Proliferation

**Figure 6:**
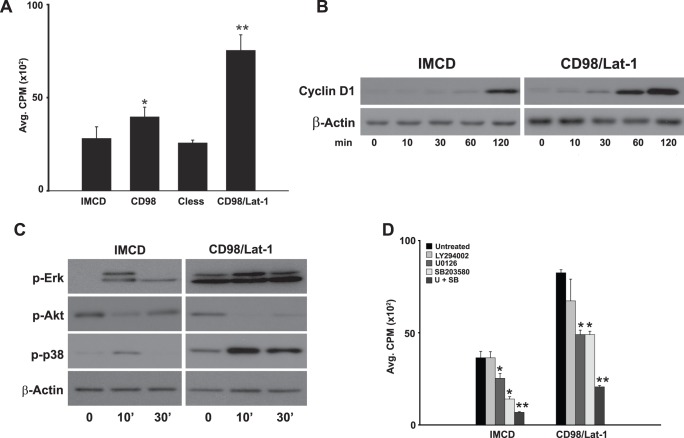
CD98hc/Lat-1-dependent amino acids transport increases cell proliferation mediated by Erk- and p38 MAPK. (A) CD98hc/Lat-1-dependent amino acids transport increases cell proliferation. Cells were plated onto 96-well plates, serum deprived for 24 hours then deprived of amino acids for 2 hours. ^3^H-Thymidine was then added in serum free media with a full complement of amino acids for an 8 hour pulse. ^3^H-Thymidine incorporation was determined as described in Experimental Procedures. Values are the mean ± s.e.m. of a representative experiment performed in eight replicates. (*)indicates statistically significant differences (p<0.05) compared to IMCD cells. (**) denotes a statistically significant difference between CD98 and CD98/Lat-1. (B, C) treatment of CD98/Lat-1 cells with amino acids increases cyclin D1 expression and MAPK activation. Cells were serum deprived for 24 hours then subjected to amino acid deprivation for an additional two hours (time 0). Cells were then treated with serum free media containing a full complement of amino acids and lysates were isolated at the time points indicated. Lysates were analyzed by SDS-PAGE and immunoblots were performed with the antibodies against cyclin D1 (B) and Erk, Akt and p38 MAPK. β-Actin is a loading control (C). (D) MAPK increases CD98/Lat-1 dependent amino acid induced proliferation. Cells were plated onto 96-well plates, serum deprived for 24 hours then deprived of amino acids for an additional 2 hours and pretreated with inhibitors prior to addition of ^3^H-Thymidine in serum free media with a full complement of amino acids for an 8 hour pulse. ^3^H-Thymidine incorporation was determined as described in Experimental Procedures. Values are the mean ± s.e.m. of a representative experiment performed in eight replicates. (*) indicates statistically significant differences (p<0.05) compared to untreated cells. U+SB denotes concomitant treatment with the MEK inhibitor U0126 (U) the p38 MAPK inhibitor SB203580 (SB). (**) denotes statistically significant differences between U+SB versus either inhibitor alone.

Due to the observation that overexpression of CD98 increased proliferation of cells grown in complete medium with serum by increasing amino acid transport, we developed conditions (see methods) to define the mechanisms whereby CD98 regulates proliferation in the absence of serum. When CD98 cells were placed in DMEM that did not contain serum they proliferated 60% more than IMCD cells as measured by thymidine incorporation (Figure 6A). This increase in proliferation was not seen in the Cless cells; but proliferation was more than 3 fold and double in the CD98/Lat-1 cells when compared to IMCD and CD98 cells respectively. Consistent with the markedly increased proliferation of the CD98/Lat-1 cells there was increased expression of cyclin D1 when compared to IMCD cells (Figure 6B). Thus CD98 significantly increases cell proliferation in the setting of exposure only to amino acids, confirming this as a mechanism of regulating cell proliferation.

We next investigated which pathways regulated CD98 dependent cell proliferation in response to amino acids. We initially defined the roles of Erk, Akt and p38 MAPK signaling by serum and amino acid starving IMCD and CD98/Lat-1 cells which were then exposed only to amino acids. Similar to the results seen when the cells were exposed to complete medium with serum, increased Erk and p38 MAPK but not Akt signaling was seen in the CD98/Lat-1 compared to the control IMCD cells (Figure 6C). We tested the role of these pathways in increasing the proliferation of the CD98/Lat-1 cells by exposing them to specific inhibitors of these pathways. As shown in Figure 6D, inhibiting MEK and p38 MAPK significantly decreases cell proliferation in both cell populations, while the PI3 kinase inhibitors have no effect. Furthermore, there was an additive effect of the MEK and p38 MAPK inhibitors, suggesting that these pathways acted synergistically to support CD98/Lat-1 cell proliferation.

### Increased Amino Acid Transport by CD98 Induces Activation of the mTor Pathway; however this Pathway is not Required for Enhanced Cell Proliferation

**Figure 7:**
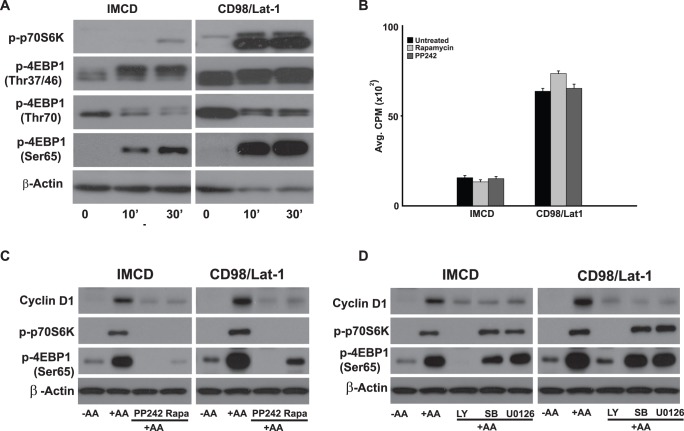
mTOR pathway is activated by amino acids but does not affect amino acid-dependent proliferation. (A) Amino acid treatment increases mTor signaling in CD98/Lat-1 cells. Cells were serum deprived for 24 hours then subjected to amino acid deprivation for an additional two hours (time 0). Cells were then treated with serum free media containing a full complement of amino acids. Lysates were isolated at the time points indicated and subjected to SDS-PAGE. Immunoblots were probed with phos-p70S6K (Thr389) as well as antibodies recognizing the multiple phosphorylated forms of 4EBP1. (B) mTor pathways do not regulate amino acid-dependent proliferation of CD98/Lat-1 cells. Cells were plated onto 96-well plates, serum deprived for 24 hours then deprived of amino acids for an additional 2 hours and pretreated with Rapamycin (100 nM) and PP242 (2.5 µM) prior to addition of ^3^H-Thymidine in serum free media with a full complement of amino acids for an 8 hour pulse. ^3^H-Thymidine incorporation was determined as described in Experimental Procedures then. Values are the mean ± s.e.m. of a representative experiment performed in eight replicates. (C and D) Inhibitors of mTor, MAPK and PI3 kinase were effective in inhibiting amino acid-dependent signaling. (C) Cells were serum deprived for 24 hours then subjected to amino acid deprivation for an additional two hours (time 0) and pretreated with Rapamycin and PP242 . Cells were treated with serum free media containing a full complement of amino acids in the presence of inhibitors. Lysates were isolated after 2hours of amino acid treatment and subjected to SDS-PAGE. Immunoblots were probed with phos-p70S6K (Thr389) as well as an antibody recognizing the hyperphosphorylated forms of 4EBP1. (D) Cells were treated as described in C in the presence of LY294002, SB 203580 and U0126, all at 10 µM. Lysates were isolated after two hours of amino acid treatment in the presence of inhibitors and subjected to SDS-PAGE.

Because of the effects of amino acids on the mTor pathway, we defined the effects of medium containing only amino acids on this pathway in CD98/Lat-1 cells. As shown in figure 7A there were markedly increased levels of activation of p70S6K in the CD98/Lat-1 cells. Consistent with this observation there was strikingly elevated phosphorylation of the slower migrating forms of 4EBP1.Taken together, these results confirm that the mTor pathway is dramatically activated when the CD98/Lat-1 cells are exposed to amino acids.

We next defined the role of this highly activated pathway in inducing the increased proliferation of the CD98/Lat-1 cells in serum free medium by growing them in the presence of Rapamycin and PP242 (Figure 7B). Addition of either of these inhibitors did not alter CD98/Lat-1 cell proliferation, despite the fact that they inhibited phosphorylation of the downstream targets of mTor, p70S6K and 4EBP1 (Figure 7C). The PI3 kinase inhibitor LY294002, which is also an inhibitor of mTor did not alter cell proliferation although it significantly decreased amino acid activation of the mTor pathway. By contrast, the MEK and p38 MAPK inhibitors which inhibit cell proliferation do not alter activation of the mTor pathway (Figure 7D). Thus although overexpression of CD98 leads to increased amino acid transport and activation of the mTor pathway, it does not play a role in cell proliferation.

## Discussion

**Figure 8:**
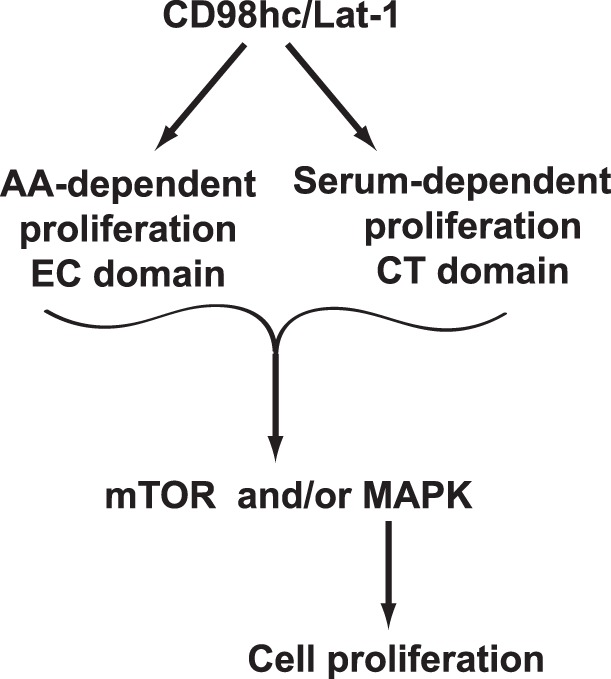
Schematic diagram demonstrating how CD98 regulates cell proliferation. Overexpression of CD98 results in increased cell proliferation which is dependent on MAPK signaling mediated by the cytoplasmic (CT) domain and increases amino acid transport which is dependent on the extracellular (EC) domain. CD98 overexpression increases mTOR signaling but this has no effect on cell proliferation.

A key function of the scaffolding protein, CD98hc, is to regulate cell proliferation. In this manuscript we demonstrate that CD98hc is required for the survival of polarized renal collecting duct cells and that it promotes cell proliferation by two distinct mechanisms. The CD98hc cytoplasmic tail promotes IMCD cell proliferation in response to complete medium with serum by activating Erk and p38 MAPK signaling. In addition, the extracellular domain of CD98hc promotes cell proliferation by increasing amino acid transport which activates the same signaling pathways. Although increased amino transport stimulates mTor signaling, this does not alter cell proliferation (Figure 8). These data clearly show that in polarized renal epithelial cells, the cytoplasmic and extracellular domains of CD98hc regulate cell proliferation by distinct mechanisms that are mediated by common signaling pathways.

We demonstrate that knocking down CD98 by siRNA or genetically deleting it from CD98^flox/flox^ collecting duct cells resulted in their death, which confirmed that CD98 is required for cell survival. These results are consistent with the early embryonic (E3.5 to 9) lethality of CD98 null mice [6]. The severe phenotype in IMCD cells lacking CD98 is likely not only due to its ability to interact with β1 integrin as β1-null collecting duct cells survive and proliferate [37]. We cannot exclude the possibility that CD98/β3 integrin interactions are sufficient for CD98 to regulate proliferation in these cells because they do express β3 integrins (data not shown). A more likely explanation for the requirement of CD98hc for IMCD cell survival is that CD98 also facilitates critical aspects of amino acid transport. This is consistent with the observation that mice expressing CD98hc that lacks an extracellular domain die between E7.5 and E 9.5 [38].

CD98 is required for proliferation of B and T lymphocytes [27], [28], vascular smooth muscle cells [29] and intestinal epithelial cells [30] in the setting of different in vivo injury models. In the lymphocyte and vascular smooth muscle studies, an interaction between integrins and CD98hc were shown to be required for the proliferative response. Our deletion mutant results demonstrate that the CD98hc cytoplasmic tail is required for these proliferative responses and the critical amino acids required for signaling are distal to the membrane proximal 5 amino acids. We previously demonstrated that an interaction between CD98hc and β integrins requires either the transmembrane domain or the proximal part of the cytoplasmic tail of CD98hc [26]. Thus although an integrin/CD98hc interaction may be necessary to promote proliferation, regions more distal to the integrin interacting site of the CD98hc tail are required to mediate these effects.

We demonstrate that overexpression of CD98hc resulted in increased activation of Erk, p38 MAPK and p70S6 kinase following treatment of cells with medium containing serum; however proliferation was only dependent on activation of Erk and p38 MAPK. These observations contrast with the cell signaling pathways modulated by CD98 following integrin ligation where FAK [18], [26], PI3-kinase [18], [23], [26] and Rac [23] but not Erk [26] were activated and regulated cell adhesion, migration and transformation of different cell types. Thus CD98 controls different pathways in the setting of integrin ligation and growth factor stimulation, which in turn modulate different cell functions.

Our data clearly demonstrates that in the setting of overexpression of CD98hc, cell proliferation is regulated by the transport of amino acids in addition to mechanisms mediated by the cytoplasmic tail which may, at least in part, require the interaction of CD98hc with integrins. This effect was even more obvious when we overexpressed Lat-1, which resulted in more significant amino acid transport than only overexpressing CD98hc. These results suggest that overexpressing CD98hc increases the surface expression of the Lats in IMCD cells, with consequent increased amino acid transport and cell proliferation. This is further emphasized when we co-express both the CD98hc and Lat-1. Our results contrast with studies on lymphocytes and vascular smooth muscle where only the interaction with integrins was required for the increased proliferative response. However they are consistent with multiple other studies where cellular proliferation is affected when the Lat amino acid transporters are overexpressed, decreased or when their function is inhibited [39], [40]. A potential reason for the differences in our findings and those in the lymphocytes and vascular smooth muscle cells are that our studies are performed on cells where CD98 is overexpressed, while those are on cells where CD98hc is deleted. Furthermore epithelial cells in the kidney have some of the highest expression levels of CD98 in the body which may potentiate their importance for cell proliferation and survival.

Increased Erk and p38 MAPK but not Akt activation was observed in serum starved CD98/Lat-1 cells treated with amino acids. It was surprising there was no increase in Akt activation, as it was recently reported that amino acids can activate mTORC2 in a PI3K/Akt-dependent manner [41]. Furthermore, this result effectively rules out the involvement of mTORC2 in the proliferative response of CD98/Lat-1 cells. A marked increase in Erk was present in the amino acid deprived CD98/Lat-1 cells. This is one of the key mechanisms whereby cells can adapt to stress induced by amino acid deprivation as sustained Erk activation increases p21(cip1/waf1) which blocks cell cycle progression and contributes to activation of the GCN2-eIF2α-ATF4 survival pathway [42]–[44]. Erk phosphorylation was increased when CD98/Lat-1 and IMCD cells were treated with amino acids and this was accompanied by increased p38 MAPK signaling. Inhibiting these pathways individually decreased cell proliferation and there was an additive effect of inhibiting both pathways together, suggesting that they activate proliferation independently from each other. Amino acid-dependent Erk and p38 MAPK activation has been shown to alter signaling of the MAPK-activated protein kinases such as p90 ribosomal S6 kinase (RSK), mitogen-and-stress-activated kinase (MSK) and possibly MAPK-interacting kinases (MNK) [45], [46]. Because these multifunctional kinases regulate numerous cellular processes including cell proliferation, this is the likely mechanism whereby CD98-dependent amino acid transport regulates cell proliferation in collecting duct cells.

We originally hypothesized that the increased proliferation in CD98/Lat-1 cells, where there was markedly increased amino acid transport, would lead to activation of the mTOR pathway. As expected when starved IMCD cells were treated with serum or amino acids, there was phosphorylation of the two best characterized targets of mTOR, 4EBP1 and S6K1 and this was markedly increased in CD98/Lat-1 cells. These effects were readily reversed by Rapamycin and the mTOR kinase antagonist PP242 a more effective ATP-competitive inhibitor that targets both mTORC1 and mTORC2 [47]. In addition, phosphorylation of both 4EBP1 and S6K1 was inhibited by the PI3K inhibitor LY294002 that is now known to inhibit mTOR as well as class III PI3K hVps34 [48], [49]. However, despite marked activation of the mTOR pathway, inhibiting it did not alter cell proliferation. Thus only the Erk and p38 MAPK pathways are important for the proliferative response of the CD98/Lat-1 IMCD cells.

Cyclin D1 was undetectable in amino acid deprived cells but its expression was restored upon amino acid treatment with an earlier recovery in CD98/Lat-1 cells. By contrast, inhibitor studies showed that Cyclin D1 was regulated by all three pathways activated by amino acid treatment. Treatment with Rapamycin, PP242 as well as LY294002 led to drastic inhibition of Cyclin D1 expression, although as mentioned previously, these inhibitors had no effect on cell proliferation following amino acid supplementation. On the other hand, treatment with the MEK inhibitor U0126 and the p38 MAPK inhibitor SB203580 effectively suppressed Cyclin D1 expression as well as inhibiting proliferation. Cyclin D1 is a key target of amino acids in hepatocytes and its overexpression can overcome cell cycle arrest induced by non-essential amino acid deprivation under mitogenic conditions [50]. Several studies have suggested that Cyclin D1 can induce cell growth and protein synthesis in addition to its well-known effects on proliferation [51], [52]. In MCF7 cells, 4EBP1 was described as a key modulator of Cyclin D1 therefore coupling its expression to mTORC1 signaling [53]. Although Cyclin D1 is downstream of all three pathways, it does not seem to be a rate limiting factor in the proliferative response to amino acids in our system, suggesting that Erk and p38 MAPK are affecting cell cycle progression and proliferation by controlling expression of cyclin-dependent kinase inhibitors such as p21 and p27 [44], [54], [55].

In conclusion, our studies demonstrate that overexpression of CD98hc by renal tubular epithelial cells increases cell proliferation by inducing signaling via its cytoplasmic tail and increasing CD98-dependent amino acid transport. These data have ramifications if CD98hc is viewed as a therapeutic target in oncological and immunological diseases. To this end CD98 has clearly been shown to play a role in the pathogenesis of these diseases in mice [27], [28], [30] and there is strong correlative data linking CD98 expression and tumor metastasis in humans [56]–[61].

## Material and Methods

### Antibodies and Other Reagents

The hybridoma cell line 4F2 (C13) (anti-CD98) was purchased from American Type Culture Collection (ATCC, Manassas, VA) and the anti-CD98 (4F2) antibody was purified from conditioned medium from the hybridoma cell lines by protein A affinity chromatography. We raised a polyclonal antibody to Lat-1 directed against the peptide sequence RHRKPEL found in the N-terminus of human Lat-1. This antibody has been commercialized by Capralogics **(**Hardwick, MA, USA). The specificity of this antibody was verified with another Lat-1 antibody (clone 3B11-1D, Serotec, Oxford, UK). Antibodies to phosphorylated and total Erk1/2, p38 MAPK, Akt , p70S6K, 4EBP1 and Cyclin D1 were all from Cell Signaling Technology (Beverly, MA). LY294002, U0126, SB203580, rapamycin and PP242 were all purchased from Calbiochem (EMD Biosciences, La Jolla, CA). Culture media were from Mediatech (Manassas, VA) and the selection antibiotics Geneticin and Zeocin were from Invitrogen (Carlsbad, CA). ^3^H- Isoleucine (Ile) (77 Ci/mM) was purchased from Amersham Pharmacia Biotech (Piscataway, NJ). ^3^H-Thymidine (6.7 Ci/mmol) was purchased from MP Biomedicals (Irvine, CA).

### Plasmids and Cell Lines

Inner medullary collecting duct (IMCD) cells, which have been previously described, were used in all experiments [62]. IMCD cells expressing either CD98 or deletion mutants of CD98 (CD98-77, CD98-82 and CD98-87) were previously described [26]. The CD98^fl/fl^ IMCD cells were isolated from CD98hc floxed mice as described previously [63]. These cells are maintained in DMEM and 10% fetal calf serum. Established cells lines were transfected with a construct expressing full length human CD98 (hCD98) or vector only. Cells expressing hCD98 were selected using flow cytometry. IMCD cells expressing the Cless mutant (kindly donated by Dr. M. Palacin (Universitat de Barcelona)) [36], [64] were generated by transfecting IMCD cells using Lipofectamine Plus (Invitrogen) with the Cless construct after which the cells were selected by adding Geneticin (G418 sulfate) (Invitrogen, Carlsbad, CA) at 1 mg/ml to the medium. Stable cell populations of cells expressing equal levels of Cless, CD98 and the mutants of CD98 were established utilizing flow cytometry under sterile conditions.

Human Lat-1 cDNA was a generous gift from Dr. Francois Verrey (University of Zurich). Cells overexpressing Lat-1 were made by transfecting with the construct, after which they were selected with Zeocin (400 µg/ml) and cloned. Multiple clones were initially made and then transfected with CD98 or the mutant CD98 constructs as described above and sorted to equal levels of expression by flow cytometry.

### Cell Survival Studies

Subconfluent monolayers (30–50%) of CD98^fl/fl^ or CD98^fl/fl^ IMCD cells reconstituted with hCD98 were infected with adeno-Cre for 16 hours. Lysates were isolated at 24 hr intervals following infection and run on 10–15% SDS gels. Immunoblots were probed with specific antibodies to human CD98 (Santa Cruz,C-20) or mouse CD98 ( Santa Cruz, M-20) as well as cleaved Caspase-3 (Cell Signaling Technology, MA) and Actin as a loading control (Sigma-Aldrich).

### Amino Acid Transport

The amino acid studies were performed as previously described [17]. The wild type or transfected IMCD cells were rinsed with warm Na^+^-free Hanks’ buffered salt solution (HBSS) (136.6 mm Choline Chloride, 5.4 mm KCl, 4.2 mm NaHCO_3_, 2.7 mm Na_2_HPO_4_, 1 mmCaCl_2_, 0.5 mm MgCl_2_, 0.44 mm KH_2_PO_4_, 0.41 mmMgSO_4_, pH 7.8), in which the sodium-containing salts were iso-osmotically replaced with choline, to remove extracellular Na^+^ and amino acids. Cells were equilibrated in warm choline-substituted HBSS for 10 min. The uptake of radiolabeled amino acids (2 µCi of [^3^H]Ile/ml) at 100 µmol/liter in 1 ml of choline-substituted HBSS was measured for 20 s at 37°C. Uptake of [^3^H]Ile was linearly dependent on incubation time up to at least 3 min. Uptake was terminated by washing the cells rapidly four times with 1 ml/well of ice-cold HBSS. The cells were lysed overnight with 1 ml 0.2%SDS/2 ml NaOH. A 0.95-ml aliquot from each well was mixed with scintillation fluid, and radioactivity was quantified in a Beckman LS 6000SC liquid scintillation counter. The remaining 0.1 ml was analyzed for protein content using a BCA protein assay reagent.

### Cell Proliferation Assay

3×10^3^ cells were plated per well in 96-well plates and maintained in DMEM/F12 devoid of serum for 24 hours prior to the experiment. When studying serum-dependent proliferation, ^3^H-Thymidine was added at 0.5 µCi/well in DMEM/F12 containing 1% FBS for up to 16 hours. When studying the effect of amino acids on proliferation, cells were incubated in DPBS containing glucose (3 g/L) and Ca^++^/Mg^++^ for 2 hours prior to the 8 hour ^3^H-Thymidine pulse. When needed, pretreatment with inhibitors was performed for up to an hour prior to the assay. The cells were then washed repeatedly with PBS then 10% Trichloroacetic Acid, solubilized with 1% SDS and radioactivity was measured using a liquid scintillation counter (Beckman, CA).

### Membrane Localization of CD98hc

To verify the proper compartmentalization of CD98 in cells transfected with the deletion constructs, the cells were fractionated into Triton X-100–soluble and –insoluble fractions, as described previously [65]. Briefly, cells were scraped in cell lysis buffer (10 mM HEPES, pH 7.2, 1% Triton X-100, 100 mM NaCl, 2 mM EDTA supplemented with protease and phosphatase inhibitors), incubated at 4°C for 20 min, followed by centrifugation at 10,000 × g for 20 min. The resulting supernatant was considered the Triton X-100/detergent-soluble fraction. For the insoluble fraction, the pellet was dissolved in solubilization buffer (10 mM HEPES, pH 7.2, 1% SDS, 100 mM NaCl, 2 mM EDTA supplemented with protease and phosphatase inhibitors), and the proteins were released by sonication. The resulting preparation was incubated at 4°C for 20 min, followed by centrifugation at 10,000 × g for 20 min. The resulting supernatant was considered the TX-100/detergent-insoluble fraction. 10 µg of each fraction was analyzed by Western blot using anti-hCD98 antibody.

### Amino Acid Deprivation Studie

Cells were serum deprived for 24 hours, rinsed repeatedly with PBS then incubated for 2 hours in PBS containing Ca^++^/Mg^++^ and glucose at a concentration identical to that in DMEM/F12 (3 g/L). Serum free DMEM/F12 was then added to cells at different time points after which they were harvested for different assays. Pretreatment with inhibitors was performed prior to being treated with DMEM/F12. After establishing dose-response curves, inhibitors were used at the following concentrations: the MEK inhibitor U0126 10 µM, the p38 MAPK inhibitor SB203580 10 µM, the PI3K inhibitor LY294002 10 µM, the mTOR pathway inhibitor Rapamycin 100 nM and the mTOR kinase inhibitor PP242 2.5 µM.

### Immunoblotting

Cells were serum starved overnight then stimulated with 10% FBS or subjected to amino acid deprivation as described above then lysed in cell lysis buffer (Cell Signaling Technology, MA) supplemented with protease and phosphatase inhibitors (Sigma, MO) at the time points indicated. After protein quantification total cell lysates (5–30 ug/lane) were run on 10–15% SDS gels, transferred to PVDF membranes (BioRad, CA) and blocked with 5% milk in TBS-T. Immunoblotting was performed with primary antibodies in 3%BSA in TBS-T then HRP-conjugated secondary antibodies in 5% milk/TBS-T and visualized using the ECL Plus western blotting detection system.

### Immunoprecipitation

Cells overexpressing CD98 or CD98 deletion mutants as well as Flag-tagged human Lat-1 were lysed in a Triton X-100 based cell lysis buffer (Cell Signaling Technology, MA) supplemented with protease and phosphatase inhibitors. Pre-cleared protein lysates were incubated overnight with a human specific CD98 antibody (ATCC, Hybridoma cell line 4F2 C13) and Protein G sepharose beads (GE Healthcare) and run on a 10% SDS gel under both non-reduced and reduced (1 mM DTT) conditions. Immunoblots were probed with human specific CD98 antibody (Santa Cruz) as well as Flag antibody (Sigma, MO).

### Statistics

The Student’s *t*-test was used for comparisons between two groups and analysis of variance using Sigma Stat software was used for statistical differences between multiple groups. *P*<0.05 was considered statistically significant.
